# Shining a light on brain tumors: a review of Raman spectroscopy in the operating room

**DOI:** 10.1117/1.JBO.31.9.090901

**Published:** 2026-09-05

**Authors:** Samuel Cutfield, Katherine Ember, Clinton Turner, Jason Correia, Kevin Petrecca, Michel Nieuwoudt, Michael Dragunow, Claude Aguergaray, Frédéric Leblond

**Affiliations:** a The University of Auckland, Centre for Brain Research Freemasons Neurosurgery Research Unit, Auckland, New Zealand; b Auckland City Hospital, Department of Neurosurgery, Auckland, New Zealand; c University of Otago, The Dodd-Walls Centre for Photonic and Quantum Technologies, Dunedin, New Zealand; d Polytechnique Montréal, Department of Engineering Physics, Montreal, Quebec, Canada; e Centre de recherche du Centre hospitalier de l’Université de Montréal, Montreal, Quebec, Canada; f Auckland City Hospital, Anatomical Pathology, Pathology and Laboratory Medicine, Auckland, New Zealand; g McGill University, Montreal Neurological Institute-Hospital, Montreal, Quebec, Canada; h The University of Auckland, School of Chemical Sciences, Auckland, New Zealand; i The University of Auckland, Department of Pharmacology, Auckland, New Zealand; j The University of Auckland, Physics Department, Auckland, New Zealand

**Keywords:** Raman spectroscopy, glioma, intraoperative diagnosis, molecular diagnostics, neurosurgery

## Abstract

**Significance:**

Maximizing brain tumor resection while preserving neurological function remains a neurosurgical challenge. Current intraoperative tools, including MRI, ultrasound, and frozen section, disrupt workflow and provide limited real-time molecular information.

**Aim:**

This review synthesizes evidence for Raman spectroscopy as an intraoperative tool in glioma and brain metastasis surgery, comparing it against established alternatives and framing findings within a diagnostic hierarchy from analytic validity through clinical utility to outcome benefit.

**Approach:**

Evidence is arranged by surgical setting: *in vivo* handheld fiber-optic probes and *ex vivo* platforms including Raman microscopy, stimulated Raman scattering (SRS) imaging, and visible resonance Raman.

**Results:**

*In vivo* multicenter studies report >85% per-measurement sensitivity and specificity for tumor versus normal classification, with nondestructive analysis in ∼2  s. Molecular marker detection, including IDH mutation status, has been demonstrated predominantly *ex vivo*. *Ex vivo* SRS generates histology-quality images and can classify selected molecular alterations within ∼90  s, though this molecular capability is not clinically validated.

**Conclusions:**

While multicenter studies support the analytic validity of Raman-based tissue detection, clinical adoption requires prospective outcome trials, standardized acquisition protocols, and regulatory advancement.

## 1 Clinical Context and Unmet Needs

### 1.1 The Burden of Brain Tumors

Over 300,000 people are diagnosed every year with brain and other central nervous system (CNS) cancer globally, with mortality remaining persistently high.^1^ The five-year survival rate for primary brain and CNS malignant tumors is only 36%.^2^ Tumors may be surrounded by or dispersed within functional healthy brain tissue. Neurosurgeons must minimize damage to healthy brain tissue that controls critical functions while maximizing tumor resection to limit the chance of recurrence. The diagnosis of molecular markers can also improve outcomes through tailored cancer therapy,^3^ yet obtaining this information currently requires time-consuming laboratory processes that can delay treatment decisions.

Brain metastases originate in nonbrain organs and spread hematogenously through the blood-brain barrier. Present in ∼15 to 20% of all cancer patients,^4^^,^^5^ their incidence ranges from 8.3 to 14.3 per 100,000 and appears to be increasing.^6^ This rising trend reflects both improved neuroimaging detection and advances in systemic cancer therapies that prolong survival, thereby increasing the window during which metastatic spread to the brain can occur.^6^^,^^7^ The prognosis for brain metastases varies from 3 to 46 months depending on patient and tumor factors.^8^

Gliomas represent the most common primary malignant CNS tumors, with an incidence of 5.8 per 100,000 in the United States.^9^ Gliomas are a broad group of tumors currently classified into adult-type, pediatric-type, and circumscribed astrocytic gliomas according to the 2021 CNS World Health Organization (WHO) classification.^10^ Based on integrated histological and molecular criteria, gliomas are assigned a CNS WHO grade within their respective tumor types, with tumors grouped as either low grade (CNS WHO grade 1 or 2) or high grade (CNS WHO grade 3 or 4). Higher grades indicate more aggressive biological features and worse prognosis, with the CNS WHO grade designed to reflect expected clinical behavior within each tumor type.^10^

### 1.2 Current WHO Classification and Molecular Markers

The classification of glioma has undergone a fundamental paradigm shift from purely histopathological criteria based on microscopic appearance to the current 2021 WHO Classification of CNS Tumors, which integrates molecular markers as fundamental diagnostic elements.^10^ This evolution recognizes that molecular signatures often predict clinical behavior more accurately than morphology alone.

Among the most important molecular markers in glioma are mutations in *isocitrate dehydrogenase* (*IDH*) *1* and *2* genes,^10^ which encode distinct but related enzymes that catalyze the same reaction.^11^ These *IDH1/2* mutations are central to the classification of adult-type gliomas into three main groups: glioblastoma (IDH-wildtype), astrocytoma (IDH-mutant), and oligodendroglioma (IDH-mutant and 1p/19q co-deleted). The molecular classification of adult-type gliomas has significant clinical and therapeutic implications, making their accurate assessment vital for optimal patient management.^12^

Glioblastoma is now exclusively defined as IDH-wildtype, representing the most prevalent and aggressive high-grade glioma, affecting 2.9 to 5 individuals per 100,000.^9^^,^^13^^,^^14^ This tumor carries the worst prognosis among all primary brain tumors. Median survival is limited to ∼9 to 14 months despite maximal multimodal therapy, including surgical resection, radiotherapy, and chemotherapy with temozolomide.^9^^,^^13^^,^^14^

Beyond IDH status, additional molecular markers influence treatment and patient prognosis, for example, O6-methylguanine-DNA methyltransferase (MGMT) promoter methylation status. This is a critical predictive biomarker in high-grade glioma that determines response to temozolomide (an alkylating chemotherapy agent) through its regulation of DNA repair enzyme expression.^15^ Approximately 40% to 65% of high-grade glioma patients have unmethylated MGMT status, conferring treatment resistance to temozolomide.^15^^,^^16^ *CDKN2A/B* homozygous deletion represents another critical marker. When present in a grade 2 or 3 IDH-mutant astrocytoma, *CDKN2A/B* homozygous deletion upgrades the tumor to a CNS WHO grade 4 and is associated with reduced median survival by more than 55%, which alters the treatment approach.^17^

Other important molecular alterations include *telomerase reverse transcriptase (TERT)* promoter mutations, *epidermal growth factor receptor (EGFR)* amplification, and specific chromosomal alterations including chromosome 7 gain and chromosome 10 loss (chr +7/−10).^10^ These markers further refine molecular classification and prognosis.

#### 1.2.1 Clinical implications for surgical decision-making

IDH-mutant gliomas are associated with better prognosis, in part due to their responsiveness to post-operative treatments like radiation and chemotherapy. Given their improved survival outcomes, surgeons may be inclined to remove as much tumor as safely possible. Taking this approach even further, some specialized centers consider supramarginal resection (removal of brain tissue beyond what appears abnormal on MRI) when it can be done without causing neurological problems. However, high-quality evidence supporting routine use of this more extensive approach remains limited.^18^^–^^20^ In contrast, IDH-wildtype glioblastomas behave more aggressively and have worse outcomes. ^21^ For these tumors, surgeons may take more cautious approaches when tumors are in or near critical brain areas that control important functions like speech or movement.

However, obtaining this molecular information currently takes days to weeks. Standard histopathological assessment with immunohistochemistry typically takes 7 to 10 days,^22^ methylation array profiling and comprehensive next-generation sequencing for mutation profiles can take 4 to 9 weeks due to real-world implementation barriers.^23^^,^^24^ This delay limits the utility of molecular information for intraoperative decision-making and may result in suboptimal surgical strategies and delayed treatment initiation. These constraints are what rapid, intraoperative molecular assessment could address.

### 1.3 Limitations of Current Tools

Neurosurgeons face numerous challenges in the surgical management of brain tumors, principally to achieve maximal safe resection while preserving neurological function. They must also navigate intricate and variable neuroanatomy while contending with the biological and structural heterogeneity of tumors. These surgical complexities are compounded by the current reliance on time-intensive laboratory analysis for critical molecular information, creating a gap in intraoperative decision-making. To address these challenges, surgeons draw on a range of established adjuncts: preoperative MRI for planning and preliminary diagnosis; intraoperative ultrasound, intraoperative MRI, and fluorescence-guided surgery to maximize resection; and frozen section for intraoperative preliminary histological diagnosis. Each has limitations related to diagnostic precision, cost or workflow disruption, and operator-dependent interpretation.

#### 1.3.1 Preoperative MRI and neuronavigation

MRI provides excellent anatomical detail and remains the cornerstone of preoperative tumor diagnostics, operative planning, and intraoperative guidance through stereotactic neuronavigation. Its role in neurosurgical oncology is supported by decades of clinical evidence;^25^ however, it has recognized limitations.

Preoperative MRI faces constraints in tissue characterization. Although the medical history of a patient often provides important clues in differentiating between high-grade gliomas and brain metastases, 5% to 15% of metastases occur without history or prior radiological evidence of cancer in other organs.^26^ For these solitary intracranial lesions, standard MRI sequences achieve <65% accuracy in distinguishing between metastases and high-grade gliomas.^27^ This accuracy can be improved with the addition of advanced techniques; diffusion-weighted imaging and perfusion sequences achieve 80% and 90%, respectively.^28^^,^^29^ By integrating multiple MRI sequences, machine learning can help predict some glioma molecular markers.^30^ However, these advanced techniques require additional imaging time, specialized software, and technical expertise, and the variability in acquisition protocols and analysis methods across institutions limits reproducibility.^28^^,^^29^^,^^31^^,^^32^ MRI also cannot detect tumor infiltration at the cellular level, with tumor tissue in glioblastoma often extending well beyond the contrast-enhancing regions.^33^ This means surgeons cannot identify areas of microscopic tumor infiltration that may lead to early recurrence.

MRI is also limited as a real-time surgical guide. Frameless stereotactic navigation is a widely used adjunct for preoperative planning and guiding tumor resection.^34^ The frameless system is registered to preoperative images and therefore loses accuracy as the brain can shift up to 10 to 24 mm during surgery. This shift arises from cerebrospinal fluid loss, tumor removal, patient positioning, and tissue retraction, so the navigated position no longer matches the actual anatomy.^35^ This progressive inaccuracy can lead to incomplete resection or damage to healthy tissue. To counter this, intraoperative ultrasound and intraoperative MRI have been increasingly adopted.

#### 1.3.2 Intraoperative ultrasound and MRI

Intraoperative ultrasound (iUS) provides imaging of the tumor or resection cavity at low cost with straightforward integration into the surgical workflow. Its live images can be used to overcome brain shift and correct for it in some navigated stereotactic systems.^36^ A 2024 meta-analysis incorporating a randomized trial reported higher rates of gross-total resection with iUS compared to neuronavigation alone in high-grade glioma.^37^^,^^38^ Image quality and interpretation are highly operator-dependent, and infiltrating tumor and surrounding edema can appear similar, so the diffuse margins of gliomas are difficult to delineate.^36^ As with MRI, it reports tissue structure rather than molecular identity.

Intraoperative MRI (iMRI) can also correct for brain shift to maximize resection extent. A meta-analysis^39^ and randomized controlled trials^40^^,^^41^ report improved gross total resection, significant for low-grade though not high-grade glioma in the pooled randomized data.^39^ However, improved resection rates have not yet translated into proven survival benefit for low-grade gliomas.^41^ While iMRI provides detailed volumetric assessment, it is limited by cost, infrastructure requirements, prolonged operative time, and surgical workflow disruption.^35^

#### 1.3.3 Fluorescence-guided surgery

Fluorescence-guided surgery with 5-aminolevulinic acid (5-ALA) is endorsed in glioma guidelines as an adjunct for high-grade gliomas.^25^ Tumor cells metabolize 5-ALA to protoporphyrin IX and fluoresce under blue-violet light to identify tissue for potential resection.^42^ In lower-grade gliomas, it is far less reliable, as these tumors frequently lack visible fluorescence.^43^ It is judged visually in routine practice, though quantitative measurement shows that fluorescence forms a continuum from imperceptible to strong rather than a clear positive or negative,^44^ and it signals tumor presence rather than molecular subtype.

Fluorescein sodium is also used for fluorescence-guided surgery. Long established in ophthalmology for retinal angiography,^45^ it is used off-label as a fluorophore in the resection of high-grade glioma and other contrast-enhancing lesions, including brain metastases.^46^ Unlike 5-ALA, it is not metabolized by tumor cells but extravasates wherever the blood-brain barrier is damaged, mirroring MRI gadolinium enhancement and providing an intraoperative equivalent of the contrast-enhancing region.^45^^,^^47^ Its signal does extend into nonenhancing tissue, though large areas of infiltrated brain remain nonfluorescent, so the full infiltrative margin is not delineated.^45^ Like 5-ALA, it is judged visually and provides no molecular-subtype information.

#### 1.3.4 Frozen section challenges

Frozen section analysis remains a valuable intraoperative tool, providing histological information that can guide surgical decision-making. It is widely available, well understood by neuropathologists, and its results directly inform established surgical decision-making. However, frozen sections have recognized limitations in real-time surgical guidance.

While frozen sections can in principle be used for margin assessment, tissue consumption, processing time of 20+ minutes per sample (including tissue preparation, sectioning, and staining), and neuropathologist availability create delays in surgical decision-making and prolonged anesthesia time.^48^ This makes extensive margin sampling impractical, particularly near eloquent regions. The rapid freezing process introduces artifacts, including ice crystal formation, compression, and obscured nuclear details that can impair diagnostic accuracy,^49^ compounded by sampling error from tumor heterogeneity.

Frozen sections cannot provide molecular profiling or mutation analysis, information that informs diagnosis and guides therapeutic decisions in neuro-oncology. This leaves a gap between what surgeons can learn intraoperatively and the molecular information that ultimately determines patient management.

#### 1.3.5 Addressing the diagnostic gap

**Fig. 1:**
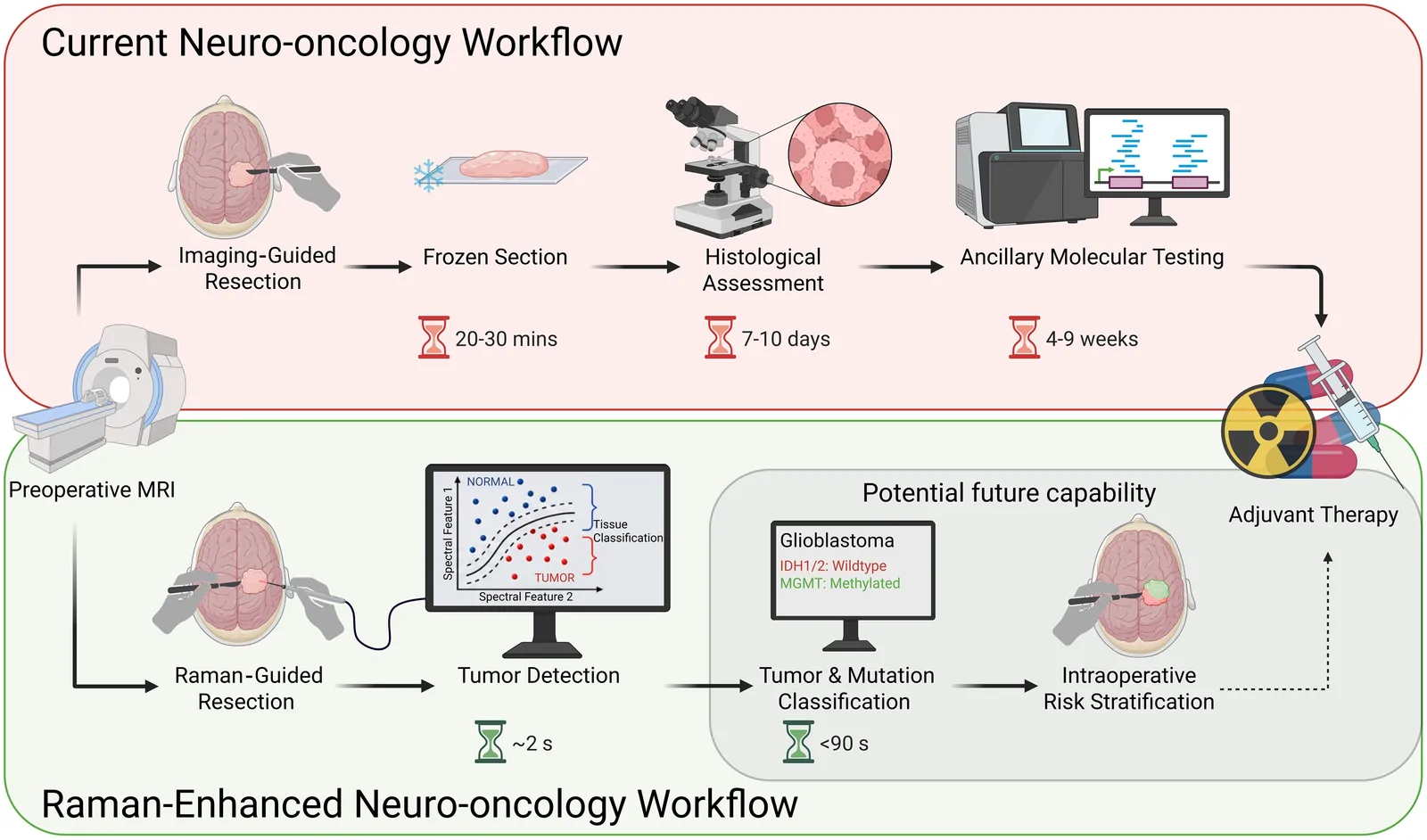
Comparative workflows for brain tumor surgery. Current standard practice (top) shows prolonged delays from surgery through to complete molecular diagnosis (up to 4 to 9 weeks), limiting intraoperative decision-making to morphological assessment via frozen section. In the Raman-enhanced workflow (bottom), spectra acquired from the resection cavity by a handheld fiber-optic probe are assigned to tumor or normal tissue classes by a trained classification algorithm, with the result returned in ∼2  s. Machine learning-based prediction of molecular alterations (<90  s) and risk-stratified surgical decisions are shown as a potential future capability and remain investigational. Hourglass icons indicate time requirements at selected stages. Created in BioRender.^50^

In the absence of real-time intraoperative diagnostic and molecular insight, surgeons often must adopt a standardized approach focused on maximal safe resection, potentially missing opportunities for more tailored interventions based on tumor biology. Raman spectroscopy, a laser-based molecular analysis technique, could address these gaps by providing real-time biochemical tissue characterization to inform tailored surgical strategies (Fig. 1).

A previous review in this journal examined the rise of Raman spectroscopy in neurosurgery through early 2020.^51^ Since that publication, the field has continued to advance. Multicenter clinical validation of *in vivo* handheld probe systems has been completed,^52^ and SRS-based platforms have advanced from tissue classification to rapid molecular subtyping and tumor infiltration mapping.^53^^,^^54^ Compatibility between Raman spectroscopy and 5-ALA fluorescence-guided surgery has been demonstrated,^55^ and early *ex vivo* spontaneous Raman spectroscopy has begun to extend from single-mutation detection toward broader molecular subtyping, so far in a small number of cohorts with limited external validation.^56^^–^^58^

The present review synthesizes these developments and evaluates the current evidence for Raman spectroscopy as an intraoperative tool, framing findings within a diagnostic evidence hierarchy and comparing the technology against established and emerging alternatives. The focus is on gliomas and brain metastases, which represent the predominant intra-axial brain tumors encountered in clinical practice. Other tumor types, including meningiomas, pituitary adenomas, and primary CNS lymphomas, are also potential targets for Raman spectroscopy but are beyond the scope of this article, although relevant technology developed in these settings is referenced where it informs the future application of Raman spectroscopy.

## 2 Raman Spectroscopy: Technical Foundation

### 2.1 Fundamental Principles

Raman spectroscopy is a laser-based analytical technique that probes the molecular composition of biological samples, with numerous forms developed as diagnostic tools for brain tissue analysis.^51^ The technique has been applied across organs,^52^ tissues,^59^ or cells.^60^ When laser light interacts with tissue, the majority of incident photons are either reflected, absorbed, or undergo elastic (Rayleigh) scattering without an energy shift. A small fraction undergoes inelastic (Raman) scattering.

Inelastic Raman scattering changes the vibrational energy of the molecule. Stokes scattering (photon loses energy) dominates at clinical temperatures; anti-Stokes scattering (photon gains energy) is weaker and rarely clinically used. The wavelength shift between incident and scattered photons depends on the atoms and bonding involved, providing a molecular identifier.^61^

A Raman spectrum plots intensity against Raman shift, measured in wave numbers (${\mathrm{cm}}^{-1}$). Each peak corresponds to a specific molecular vibration.^62^ Together, these peaks form a molecular fingerprint that identifies tissue components without reagents or tissue destruction. In complex biological samples, the spectra of similar molecules can overlap, presenting analytical challenges. However, molecular families (proteins, lipids, and nucleic acids) can be differentiated by their characteristic spectral peaks, supporting semi-quantitative comparison of tissue molecular composition. This “spectral fingerprinting” approach has supported cancer detection across multiple locations, including lung,^63^ breast,^64^^,^^65^ and prostate.^66^^,^^67^ These applications provide the rationale for evaluating Raman spectroscopy in neurosurgery.

#### 2.1.1 Technical variants

The most used approach, spontaneous Raman spectroscopy, uses a single monochromatic continuous-wave laser to illuminate the sample without preparation. Inelastically scattered light is collected by a spectrometer that diffracts it onto a detector, usually a charge-coupled device (CCD). The resulting spectral signatures are paired with chemometric or machine-learning classifiers to characterize tissue, with deep-learning methods now widely used in this role.

Coherent Raman techniques, coherent anti-Stokes Raman scattering (CARS) and stimulated Raman scattering (SRS), use two synchronized laser beams tuned to a specific vibrational frequency. Coherent excitation amplifies the signal by orders of magnitude over spontaneous Raman and supports label-free imaging at microscopic resolution. This makes CARS and SRS well suited to *ex vivo* tissue imaging, at the cost of more complex instrumentation.^68^^,^^69^

Two further enhancement mechanisms have been developed. Resonance Raman scattering uses excitation wavelengths tuned to a target molecule’s electronic absorption band, amplifying its Raman signal by $10^{2}$ to $10^{4}$ and allowing detection of endogenous chromophores.^70^ Chromophore selectivity depends on the excitation wavelength, with visible (532 nm) excitation, termed visible resonance Raman (VRR), amplifying carotenoid bands.^70^^,^^71^ Surface-enhanced Raman scattering (SERS) uses plasmonic gold or silver nanostructures to amplify the local electromagnetic field at the metal surface, enhancing the Raman signal of adsorbed molecules by $10^{6}$ to $10^{10}$ and enabling trace-level detection.^72^

### 2.2 Molecular Biomarkers in Brain Tissue

#### 2.2.1 Raman biomarkers for brain cancer

The unique Raman spectral signatures of brain tissue reflect its complex molecular composition, with specific spectral features (from amino acids, lipids, nucleic acids, and proteins) distinguishing tumor from normal brain and correlating with tumor grade and molecular subtype.^73^^,^^74^ When analyzed using predictive algorithms, these spectral features enable the classification of tumors based on their molecular features.

In gliomas and metastases, Raman spectra typically show elevated protein/phenylalanine bands alongside reduced lipid-associated peaks relative to normal brain.^59^^,^^70^^,^^75^ These features reflect the hypercellularity, extracellular matrix remodeling, and lower myelin (lipid) content of tumor tissue compared to normal brain, with findings corroborated by mass spectrometry and infrared spectroscopy.^76^^,^^77^ Translating to clinical applications, these spectral biomarkers allow intraoperative Raman systems to detect residual tumor at surgical margins by distinguishing the protein-rich, lipid-poor signature of infiltrating glioma from normal parenchyma.^75^

Raman markers also correlate with tumor grade. Relative to lower grades, high-grade gliomas show a shift in protein secondary structure from α-helix to β-sheet using VRR, and rising tryptophan signals that decline again in glioblastoma as apoptosis and necrosis reduce tryptophan content,^70^ along with enhanced nucleic acid signals consistent with increased DNA turnover.^78^ The lipid-to-protein intensity ratio in the high-wave-number region falls progressively from normal brain to low- and high-grade glioma.^70^^,^^78^

#### 2.2.2 Mutation and methylation molecular signatures

Raman spectroscopy does not directly detect genetic mutations or epigenetic modifications; it detects downstream biochemical changes that correlate with specific genetic alterations. For example, IDH-mutant gliomas accumulate the oncometabolite D-2-hydroxyglutarate. The most consistent spectral finding is an increased nucleic acid (DNA) signal,^58^^,^^79^ accompanied by reduced collagen.^57^ Lipid and cholesterol changes are less consistent: some studies report reductions in IDH-mutant tumors,^57^^,^^79^ whereas others report increased cholesteryl-ester signals in both fresh and formalin-fixed tissue, with the latter shown not to be an artifact of formalin-fixed paraffin-embedded (FFPE) processing.^56^^,^^58^

MGMT promoter methylation status correlates with distinctive lipid profiles detectable by label-free Raman microspectroscopy. Unmethylated (temozolomide-resistant) gliomas exhibit upregulated lipid oxidation/efflux and downregulated lipid synthesis pathways.^80^ The remaining markers in Table 1 (1p/19q codeletion and the exploratory TERT, EGFR, CDKN2A/B, and chromosome 7/10 alterations) have tentative band assignments from a deep-learning saliency analysis in a single probe-based Raman study, but these lack external validation, and the bands are not marker-specific.^57^

In summary, Raman spectroscopy detects molecular signatures ranging from bulk protein and lipid differences to mutation-associated metabolic profiles (Table 1). These support two potential roles in neurosurgery: tissue discrimination for surgical guidance, which relies on the larger protein and lipid differences, and molecular characterization, which relies on the subtler correlates of genetic alterations. Because this molecular characterization is indirect, its reliability depends on the strength and consistency of the biochemical-genetic correlation. The clinical performance of these markers is examined in Sec. 3.

**Table 1:** Glioma molecular markers and their contributory Raman features.

Molecular marker	Associated tumor type	Associated biochemical change	Reported Contributory Raman features (${\mathrm{cm}}^{-1}$)^a^	Evidence status
*IDH1/2* mutation	IDH-mutant astrocytoma and oligodendroglioma	Increased nucleic-acid (DNA) signal; altered phospholipid profile and reduced collagen downstream of D-2-hydroxyglutarate; lipid and cholesterol changes reported but inconsistent in direction	↑ DNA ∼498/826/1093;,^79^ ∼1342 to 1376,^58^ ↑ phosphatidylinositol ∼577, ↑ phospholipid ∼1078, ↓ collagen ∼1448;^57^ cholesteryl-ester bands ∼419 to 430 and ∼532 reported elevated^56^^,^^58^	Most validated; ∼87% to 91% *ex vivo*^57^^,^^58^^,^^79^
MGMT promoter methylation	Glioblastoma	Lipid-droplet cholesteryl-ester accumulation (higher when methylated); downregulated lipid-efflux genes	↑ cholesteryl ester ∼702 & ∼1742;^80^ ↑ protein ∼951/1560, ↓ fatty acid ∼1442^57^	Preliminary; degraded on external validation;^57^ single-institution mechanistic support^80^
1p/19q codeletion	Oligodendroglioma	Amino-acid and nucleic-acid spectral differences; also detected by SRH/AI	↑ amino acid (serine/cysteine) ∼524, ↑ nucleic acid ∼1085^57^	Retained on external validation; ∼85% to 94%^53^^,^^57^
*ATRX* loss	Astrocytoma	No direct molecular correlate; histoarchitectural SRH features classified by AI	No marker-specific band (SRH morphology; SRS 2845/2930 channels nonspecific)	∼91% by SRH + AI^53^
*TERT*, *EGFR*, *CDKN2A/B*, chr+7/−10	Typical of GBM; TERT also in oligodendroglioma, CDKN2A/B loss in astrocytoma	Minor protein/lipid differences; biochemical basis not established	No validated peaks (classifier-weighted only)	Exploratory; degraded on external validation^57^

a ↑/↓ indicate the direction of association (increase or decrease) of each band with the marker, from classifier-saliency^57^ or abundance/discriminative analysis;^56^^,^^80^ they show direction, not quantification. Listed bands are contributory features, not validated stand-alone biomarkers; classification relies on whole-spectrum machine learning, not single bands. The $∼702/∼1742\;{\mathrm{cm}}^{-1}$ cholesteryl-ester bands are from an MGMT study in glioblastoma.^80^ The $2845/2930\;{\mathrm{cm}}^{-1}$ SRS channels are common to all SRS/SRH-based detection, not marker specific.^53^

## 3 Clinical Implementation Platforms

**Fig. 2:**
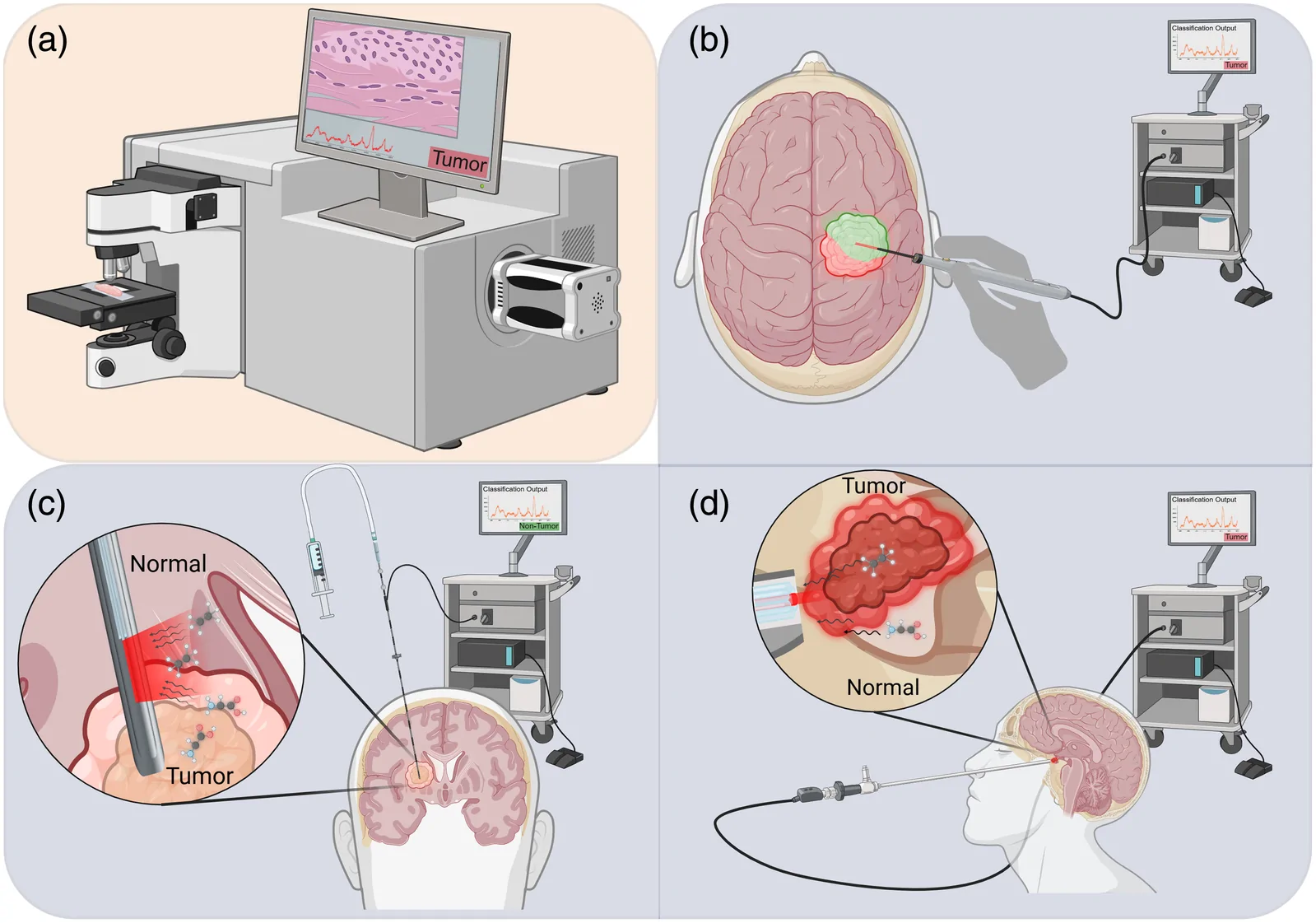
*Ex vivo* and *in vivo* surgical implementations of Raman spectroscopy in neuro-oncology. (a) *Ex vivo* SRS microscopy (orange background) provides histology-quality molecular analysis within 90 s. (b)–(d) *In vivo* applications (blue backgrounds) enable real-time tissue interrogation during surgery: (b) Handheld fiber-optic probe for margin assessment (∼2  s per measurement) during open craniotomy; (c) Biopsy needle with integrated Raman guidance for pre-sampling tissue characterization along the needle trajectory; (d) Endoscopic Raman for narrow surgical corridors (e.g., endonasal); a future direction for intra-axial tumor surgery. Panels (b)–(d) produce a tumor-versus-normal classification. Created in BioRender.^82^

Given the potential for nondestructive molecular sensing, researchers have been evaluating the clinical application of Raman spectroscopy in neuro-oncology since the early 2000s.^81^ There are two main ways in which Raman spectroscopy can fit into the clinical workflow (Fig. 2). *Ex vivo* systems analyze tissue samples outside the body. These samples include fresh surgical biopsies for immediate diagnostic guidance as well as archived specimens (FFPE or fresh frozen) for research and validation studies. *In vivo* systems must operate in real time during surgery, acquiring data within seconds to enable uninterrupted surgical guidance. Most Raman spectroscopy studies have been carried out on *ex vivo* biopsies, but significant developments have been made *in vivo*.

### 3.1 *Ex Vivo* Systems

Within the *ex vivo* setting, many Raman approaches have been described. Three are discussed below: spontaneous Raman (in microscope and fiber-optic probe configurations), coherent Raman imaging based on SRS, and VRR; a fourth, SERS is covered briefly as an emerging direction. Classification accuracies for the spontaneous Raman (probe and microscopy), SRS, and VRR platforms are compared in Table 2.

**Table 2:** Comparison of Raman spectroscopy platforms for intraoperative neuro-oncology.

Feature	Spontaneous Raman (Probe)	Raman Microscopy (*Ex vivo*)	SRS	VRR (Probe, visible-resonance)
Acquisition mode	*In vivo or ex vivo*, single point	*Ex vivo*, point-scan mapping	*Ex vivo*, wide-field imaging	*Ex vivo*, single point (theater-adjacent)
Acquisition time	0.2 to 2 s per point^75^	Minutes to hours per section	10 to 90 s per sample^53^^,^^54^	1 to 5 s per spectrum^71^
Spatial resolution	∼0.5 mm (probe spot)	1 to 2 μm	∼1 μm	∼0.5 mm (probe spot)
Laser configuration	Single CW laser (785 to 1064 nm)	Single CW laser (532 to 1064 nm)	Dual pulsed lasers (synchronized)	Single CW laser (532 nm)
Fiber-optic compatible	Yes	N/A (benchtop)	No (free-space optics)	Yes (handheld)
Sample preparation	None	Excised tissue (thin section or surface)	Excised tissue, fresh, ∼10 s prep	None (excised tissue)
Tumor versus normal brain detection accuracy	*In vivo:* Sens/spec>95% (metastases, meningioma); ∼85% to 91% (GBM)	∼97% (fresh);^59^ GBM 90% to 100% sensitivity, weaker on normal tissue (FFPE)^83^	FastGlioma: >90% AUROC ^54^	Accuracy>80% ^71^
Molecular marker detection	*Ex vivo*: IDH, 1p/19q, MGMT and others (preliminary)^57^	IDH (∼87% to 91%), 1p/19q (preliminary), MGMT (preliminary)^58^^,^^80^^,^^84^	DeepGlioma accuracy: IDH (∼95%), ATRX (∼91%), 1p/19q (∼94%)^53^	Not demonstrated
Fluorescence interference	Significant; NIR mitigation	Moderate; NIR mitigation	Minimal (inherently suppressed)	Higher (visible 532-nm excitation)
Regulatory status	Health Canada Investigational Testing Authorization	Research use only	Research use only	Research only
Evidence level	Prospective multicenter analytic validation (*in vivo*)^52^	Single-center analytic validation (*ex vivo*)	Prospective multicenter analytic validation (*ex vivo*)^53^^,^^54^	Analytic validation in clinical cohorts (*ex vivo*, theater-adjacent)^70^^,^^71^

Abbreviations: CW, continuous wave; NIR, near-infrared; SRS, stimulated Raman scattering; IDH, isocitrate dehydrogenase; ATRX, alpha thalassemia/mental retardation X-linked; MGMT, $O^{6}$-methylguanine-DNA methyltransferase; GBM, glioblastoma; Sens, sensitivity; Spec, specificity. SERS-based intraoperative approaches discussed in Sec. 3.1.4 remain at a single-study proof-of-concept stage in animal models with *ex vivo* human tissue validation and are not included in this platform comparison.

#### 3.1.1 Spontaneous Raman microscopy

*Ex vivo* Raman systems have moved from early technical-validation studies toward intraoperative clinical use. They provide rapid, complementary molecular information during surgery rather than replacing conventional molecular diagnostics. By analyzing fresh tissue samples within minutes of resection, *ex vivo* systems could potentially inform surgical decision-making and treatment planning.

Multiple *ex vivo* approaches have been developed, with Raman microscopes representing the most established platform. Raman microscopes combine conventional microscope optics with Raman spectroscopy to provide high spatial resolution (1 to 2  μm) molecular imaging of tissue sections. These systems can differentiate specific cell types,^85^ map tumor margins and infiltration patterns,^86^ and classify biofluid droplets for liquid biopsy applications.^87^ However, they require thin tissue sections or surface measurements, and, due to the small sampling volume, it can take minutes to hours to take measurements from a biopsy.

Early validation studies using FFPE *ex vivo* brain biopsies showed promising results, with sensitivities and specificities above 85% in differentiating glioblastoma, brain metastases, and normal brain.^83^ However, it is well recognized that FFPE tissue processing can affect spectral quality and impact prediction accuracy.^88^ This is because both formalin and paraffin have their own Raman spectral signatures.^89^^,^^90^ Formalin induces cross-links between proteins, glycoproteins, nucleic acids, and polysaccharides, which are not present in native tissues.^91^ Despite these challenges, FFPE tissue analysis remains valuable for retrospective studies and biomarker discovery when fresh tissue is unavailable.

Fresh tissue analysis has emerged as the preferred approach for *ex vivo* Raman spectroscopy, preserving native molecular signatures without processing artifacts. Research using *ex vivo* fresh tissue samples has yielded higher accuracies than those with FFPE samples.^84^^,^^88^ In such studies, freshly excised tissue is analyzed immediately after removal. This approach represents intraoperative biochemical conditions more accurately than FFPE sections. Research employing fresh tissue has demonstrated accuracies exceeding 88% in distinguishing tumor from nontumor tissue and around 90% in differentiating brain metastases from gliomas.^59^^,^^92^ These accuracies support a role for *ex vivo* Raman in intraoperative tissue classification, pending prospective outcome data.

In practice, "fresh tissue" in this literature includes both truly fresh specimens and snap-frozen specimens analyzed after thawing. Direct paired comparisons in brain tumor tissue show that cryofixation causes a largely reversible reduction in overall spectral intensity without altering peak relationships,^88^ and SRH-based analysis remains comparably reliable between fresh and defrosted samples.^93^ However, freeze artifacts in ice-damaged regions can reduce classifier accuracy by ∼20%,^94^ and a broader hierarchy of fresh > fresh-frozen > FFPE classification performance is consistently observed.^84^^,^^88^

Beyond tumor-versus-normal and tumor-type classification, *ex vivo* studies have examined whether spontaneous Raman can subclassify gliomas by genetic mutation. This work has used predominantly fresh or frozen specimens rather than FFPE tissue. Of these, IDH-mutation detection has the strongest evidence base, with independent groups reporting accuracies of ∼87% to 91% (Table 1).^57^^,^^58^^,^^79^^,^^84^

Further efforts have examined whether Raman spectroscopy can detect downstream molecular changes related to other clinically important glioma mutations. One study of 44 patients focused on deep learning-assisted Raman spectroscopy to detect seven molecular subgroups (*IDH*, 1p/19q, *MGMT*, *TERT*, *EGFR*, chr +7/−10, and *CDKN2A/B* homozygous deletion). Across the seven subgrouping tasks, the model achieved a sensitivity of 83.3% and a specificity of 85.0%.^57^ However, external validation revealed limitations, with accuracy maintained only for IDH and 1p/19q while performance was reduced for other markers. The drop in performance for some molecular subgroups was attributed to data imbalance, subgrouping instability, and minimally detectable biochemical changes; addressing these will require larger, more diverse datasets.

#### 3.1.2 Coherent Raman scattering (SRS) imaging

Coherent Raman scattering microscopy (CARS and SRS) provides rapid, label-free histological assessment of fresh brain tissue during surgery. They can be used to detect and visualize lipid-rich structures such as myelin due to the strong response to ${\mathrm{CH}}_{2}$ vibrational modes.^95^^,^^96^ They can also be used to analyze the protein-to-lipid ratios and distributions by targeting specific vibrational resonances.^96^^,^^97^ This biochemical detail supports their potential use in intraoperative guidance and neuropathology. Three SRH-based systems have been developed from the same research group. These use freshly excised tissue, so despite their intraoperative use, they are *ex vivo* techniques, and the molecular-classification platforms built on them remain investigational.

DeepGlioma is a molecular diagnostic platform that combines SRS imaging with artificial intelligence for tumor classification.^53^ The system acquires SRS images at two Raman shifts (2845 and $2930\;{\mathrm{cm}}^{-1}$) to capture lipid and protein content, generating virtual hematoxylin and eosin (H&E)-like images. Deep learning algorithms trained on labeled images analyze these images to predict molecular alterations. The entire process, from tissue placement to molecular classification, requires ∼90  s.

Clinical validation in 153 patients demonstrated high accuracy: >95% for distinguishing IDH-wildtype from IDH-mutant gliomas, >94% for detecting 1p/19q co-deletion in oligodendrogliomas, and >91% for identifying alpha thalassemia/mental retardation X-linked (ATRX) loss in astrocytomas.^53^ The system’s ability to provide near-real-time molecular classification during surgery illustrates the potential of SRS-based approaches in this setting.

FastGlioma focuses specifically on rapid margin assessment to detect residual tumor.^54^ Building on the same SRS imaging technology, FastGlioma emphasizes speed and simplicity for intraoperative decision-making. Fresh tissue samples from the resection cavity are imaged within 10 s of excision, with AI algorithms providing immediate classification of tumor infiltration with >90% area under the receiver-operating-characteristic curve (AUROC).^54^

In a recently posted preprint that has not yet been peer-reviewed, Goff et al. introduce the SIGNAL model, a 27-million-parameter SRH classifier with an attention-based mechanism that rejects artifact-laden image patches before classification. Trained on 1.56 million fields of view from 967 adult diffuse glioma patients, SIGNAL reports improved real-world accuracy over DeepGlioma for IDH mutation (93.5% versus 79.2%), 1p/19q co-deletion (93.5% versus 88.3%), and ATRX loss (89.6% versus 84.0%), with maintained classification performance in margin samples despite lower patch retention.^98^ Because these figures come from a single preprint, they should be regarded as preliminary pending independent validation.

DeepGlioma, FastGlioma, and SIGNAL use the NIO Laser Imaging System (Invenio Imaging Inc.), a stimulated Raman histology platform with international multicenter validation and in clinical use for intraoperative histology.^53^^,^^54^

These systems address different goals: DeepGlioma and SIGNAL both classify molecular markers, with SIGNAL the next iteration designed to be more robust on the uncurated tissue of surgery, whereas FastGlioma targets rapid detection of tumor infiltration at the margin. All three share a design choice worth scrutinizing: they classify or map only viable, cellular tissue and filter out blood, necrosis, and cautery damage. For reading a molecular subtype this filtering is reasonable, since the signal comes from intact tumor cells and discarding uninformative regions guards against incorrect classifications. The discarded tissue is not always uninformative to the surgeon, though. Necrosis is itself a grade-defining feature in the current WHO CNS tumor classification,^10^ and the infiltrative margin is where surgical decisions are hardest. The molecular classifiers also report only the diagnostic triad (IDH, 1p/19q, ATRX), not the wider markers that drive molecular grading, which so far only a single study has attempted, and external validation has not supported (Sec. 3.1.1). Reliable guidance across a whole tumor and its margin will ultimately need models trained on this heterogeneity, not only the cleanest samples.

#### 3.1.3 Visible resonance Raman

Zhang and colleagues reported the first use of a portable VRR analyzer (VRR-LRR) on freshly resected glioma tissue analyzed in the room adjacent to the operating theater, collecting 2220 spectra from 63 specimens taken from 52 patients. Their classifier, built using principal component analysis with a support vector machine, discriminated glioma from normal brain with greater than 80% accuracy against histopathology.^71^ Whether 532 nm excitation can be safely deployed in direct contact with intact brain at surgical power densities remains to be evaluated.

#### 3.1.4 Surface-enhanced Raman scattering (SERS)

A post-2020 SERS approach to brain-tumor surgery has demonstrated proof of principle in *ex vivo* human glioma tissue, though at a stage distinct from the established modalities above. This group described an intelligent SERS navigation system that delineates the resection margin by sensing metabolic acidosis, a feature of tumor cell metabolism.^99^ A small water droplet placed on the tissue at the resection margin collects acidic metabolites from the tumor microenvironment and is then transferred to a pH-sensitive SERS chip outside the patient. The chip Raman reporters generate a pH-dependent signal, which a deep-learning model converts into a pH map of the resection bed. Because the chip operates outside the patient, no exogenous contrast agent is administered. In glioma-bearing rats, the approach extended overall survival relative to conventional resection, and excised human glioma tissue was successfully pH-mapped, although in-human prospective clinical evaluation has not been reported.^100^

### 3.2 *In Vivo* Systems

*In vivo* Raman spectroscopy interrogates tissue in real time during surgery, without the excision that frozen section and *ex vivo* methods require.

#### 3.2.1 Handheld surgical probes: real-time guidance

Among *in vivo* Raman implementations, handheld fiber-optic probes have undergone the most extensive clinical validation through multicenter investigational trials. These pen-shaped, sterilizable devices contain sophisticated optics miniaturized to fit within the probes. The surgeon places the probe tip on or near brain tissue; the system illuminates the site, collects the Raman scattered light, and returns a tissue classification within seconds.^52^

Clinical performance has been initially validated with smaller trials, leading to more recent multicenter trials. The landmark study by Jermyn *et al.* involving 17 patients with glioma achieved a prediction accuracy, sensitivity, and specificity of over 90% in differentiating tumor from normal tissue. This included the critical capability of detecting normal brain infiltrated with glioma cells, addressing a key limitation of MRI and visual inspection.^75^

The subsequent larger multicenter study using a sterilizable handheld probe (Sentry) system, which has Health Canada Investigational Testing Authorization, provided further clinical evidence. In this study involving 67 patients across multiple institutions, neurosurgeons used the sterilizable handheld probe to obtain Raman spectra from various brain regions during tumor resection. The single-point system collects scattered light from a spot with a 0.5 mm diameter. The collected spectra were assessed based on Raman signal quality and, if adequate, used to inform a machine learning model that classifies tissue based on molecular fingerprint. The system reported sensitivity and specificity above 95% for brain metastases and meningioma and ∼85% to 91% for glioblastoma, validated against gold standard pathology.^52^ Acquisition and classification required two seconds per measurement, enabling extensive sampling without workflow disruption. These accuracy figures are reported at the measurement (per-site) level, with surgeons integrating information from multiple assessments throughout resection to guide surgical decisions.

A follow-up study from the same group identified additional tumor types (astrocytoma IDH-mutant, oligodendroglioma IDH-mutant, and 1p/19q-co-deleted; ependymoma) and showed that Raman performance was maintained during simultaneous 5-aminolevulinic acid (5-ALA) fluorescence imaging, indicating compatibility with existing surgical adjuncts. ^55^

Taken together, these studies trace a path from single-center proof of concept to multicenter validation across more institutions and tumor types; the most recent work also confirms the probe performs alongside 5-ALA fluorescence. However, two limitations remain: this multicenter *in vivo* probe research has come from a single collaboration (Montreal and New York), so its generalizability to other groups and instruments is not yet demonstrated; these studies have classified tumor types rather than predicting molecular-marker status directly. IDH-status detection has been demonstrated only on excised tissue (Sec. 3.1.1), not through an *in situ* probe.

#### 3.2.2 Integration with existing surgical tools

Beyond the handheld probe, the same detection approach is being built into instruments surgeons already use, moving Raman from a separate device toward an embedded capability. Two directions have reached early clinical testing: biopsy needles with integrated Raman optics and endoscopic probes for narrow surgical corridors.

Biopsy needle integration incorporates illumination and collection optics directly within biopsy needles, allowing tissue to be interrogated at the needle tip before sampling. Clinical testing in three patients showed that quality Raman spectra can be acquired *in situ* during brain biopsy. This proof-of-concept points to a potential role in improving diagnostic yield and reducing nondiagnostic samples, though technical challenges remain in miniaturizing the optics while maintaining signal quality.^101^

Endoscopic Raman has been developed along two technology lines. The Leblond group has built a bayonet-handled fiber-optic Raman probe sized for the endonasal corridor (2.1 mm outer diameter, 11 cm reach) and tested it *in situ* during transsphenoidal pituitary adenoma resection, later demonstrating Raman-based *ex vivo* detection of pituitary adenomas.^102^ Flexible, coherent Raman fiber endoscopes for true *in situ* imaging through narrow surgical corridors remain at the proof-of-concept stage.^103^ Neither has yet been used for intra-axial tumor surgery, but together they move endoscopic Raman from a concept demonstration into first clinical testing. A future role in neuroendoscopic tumor surgery is now plausible rather than speculative.

### 3.3 Clinical Implementation Strategy: Balancing Complexity and Capability

Raman platforms trade technological complexity against information content (Table 2). Spontaneous Raman systems offer relative simplicity with single laser sources, straightforward optics, and robust fiber-optic compatibility, making them practical for routine *in situ* surgical guidance. These systems are relatively affordable and provide immediate feedback during active resection, with full spectral acquisition enabling comprehensive molecular analysis and biomarker discovery. However, weak signals require careful fluorescence mitigation strategies and longer acquisition times for detailed mapping.

Coherent techniques (CARS and SRS) provide enhanced signals enabling video-rate imaging and superior spatial resolution for detailed tissue architecture visualization. While more complex and expensive, they offer histology-quality *ex vivo* analysis when detailed molecular information is critical. The high signal-to-noise ratios support reliable automated analysis, but these benefits require complex dual-laser systems with precise synchronization and current incompatibility with flexible fiber-optic delivery.

Future implementation could involve hybrid approaches using both technologies synergistically: *in vivo* Raman guides initial resection and identifies suspicious areas, while *ex vivo* SRS provides detailed analysis of excised samples to confirm margins and determine molecular subtypes.

A further consideration is how a neurosurgeon can interpret each system’s output. DeepGlioma, FastGlioma, and SIGNAL produce virtual H&E images a neurosurgeon or pathologist is familiar with reviewing, and SIGNAL also indicates regions it used to make its classification. By contrast, spectroscopic probes return a label from spectral features no human reads by eye, so the surgeon must trust the classification rather than assess the tissue themselves. Closing this gap will mean grounding the discriminating spectral features in the tissue biology clinicians already work with, and reporting how confident each prediction is, or flagging when a measurement falls outside the training data. An output the surgeon can interpret, with a measure of uncertainty, is more likely to be trusted than an opaque label.

## 4 Clinical Applications and Impact

When evaluating intraoperative diagnostic technologies, three levels of evidence should be considered:

• Analytic validity (can the tool accurately identify the target?)
• Clinical utility (does the information change clinical decisions?)
• Outcome benefit (do those changed decisions improve survival or function?)

This hierarchy shows accuracy alone is necessary but not sufficient to establish clinical value.^104^ The current evidence for Raman spectroscopy in neurosurgery is predominantly at the diagnostic accuracy level, with early prospective data suggesting clinical utility when combined with established adjuncts.^105^ Prospective randomized trials demonstrating that Raman-guided surgery improves patient outcomes have not yet been conducted. This distinction is important for contextualizing the findings discussed below.

### 4.1 Surgical Guidance and Margin Detection

The most validated surgical application of Raman spectroscopy is *in vivo* real-time discrimination between tumor and normal brain tissue at resection margins. This capability directly addresses one of the challenges of neurosurgery: achieving maximal safe resection when tumor boundaries are visually indistinguishable from normal brain. This is particularly important in infiltrative gliomas where tumor cells extend far beyond visible abnormalities.

Since the proof-of-concept study by Jermyn et al.,^75^ subsequent work has demonstrated *in vivo* Raman spectroscopy’s ease of use, workflow integration, speed, and applicability across tumor types.^52^^,^^55^ The potential surgical use case is margin assessment: a neurosurgeon could sample multiple points across a resection cavity, obtaining information from each site in two seconds without tissue destruction, overcoming several of the frozen section limitations discussed earlier. However, total margin assessment time, optimal sampling density, and number of points per patient have not been systematically reported in the literature, representing an important evidence gap for clinical translation. This suits locations where frozen section is not an option, such as margins near eloquent cortex, and adds little to operative time.

The addition of Raman spectroscopy to the neurosurgeon’s armamentarium could potentially improve the extent of resection, an objective shared by other intraoperative tools, including 5-ALA fluorescence imaging and intraoperative US and MRI.^35^^,^^106^ Increasing extent of resection is well documented to translate to meaningful survival benefit in glioma,^107^ with gross total resection in glioblastoma increasing patient survival at one year by 61% and two years by 19% compared to subtotal resection.^108^ However, these survival figures are derived from extent of resection studies, not from Raman-guided surgery, and whether this technology can improve resection or survival remains to be demonstrated prospectively.

#### 4.1.1 Comparison with 5-ALA fluorescence-guided surgery

5-ALA fluorescence is the most established intraoperative optical adjunct in glioma surgery and the only one with level I outcome data. A phase III randomized controlled trial demonstrated improved extent of resection and progression-free survival in glioblastoma,^106^ and its use has since been widely adopted. Any assessment of Raman spectroscopy’s potential role must therefore be considered alongside it.

Fluorescein sodium is another fluorophore used in many centers and offers practical advantages over 5-ALA, including lower cost, administration at induction of anesthesia, and no requirement for postoperative light precautions.^45^^,^^46^ Its evidence base is less established and rests on a prospective multicenter phase II study without a control group.^47^ No randomized outcome data are available. Fluorescein has not been compared directly with Raman spectroscopy, though the combination has been proposed,^45^ so the narrative comparison that follows is restricted to 5-ALA.

5-ALA has recognized limitations. Its fluorescence is subjectively assessed by the surgeon and signals tumor presence without the molecular specificity to distinguish tumor types or detect specific mutations. It also requires oral administration 2 to 4 h before surgery,^106^ and fluorescence is inconsistent in brain metastases and frequently absent in low-grade gliomas.^43^^,^^109^ Recent work pairing stimulated Raman histology and fluorescence microscopy has demonstrated that PpIX accumulates preferentially in tumor-associated macrophages, although astrocyte-like malignant cells also showed enhanced PpIX, raising questions about the cellular basis of 5-ALA fluorescence.^110^

Raman spectroscopy and 5-ALA should be viewed as synergistic rather than competing technologies. The two can be used concurrently, as shown in the multicenter probe study discussed in Sec. 3.2.1. In an *ex vivo* comparison of resection margin samples, Raman spectroscopy detected residual tumor with higher sensitivity than 5-ALA fluorescence at the resection boundary.^111^ The only prospective clinical trial directly comparing the two modalities intraoperatively found that Raman spectroscopy offered higher sensitivity in the infiltration zone while 5-ALA provided greater specificity. Their combined use increased detection accuracy by ∼10% and was associated with a 52% increase in resection volume beyond preoperative contrast-enhancing boundaries.^105^ Whether this additional resection improved survival or function was not assessed. These findings suggest Raman spectroscopy could address gaps in 5-ALA coverage, particularly at infiltrative margins, in nonfluorescent tumor subtypes, and where molecular specificity is required. Even so, 5-ALA remains the best-validated intraoperative adjunct.

### 4.2 Molecular Diagnostic Impact

The *ex vivo* evidence that spontaneous Raman and SRH-based approaches can detect clinically relevant alterations (IDH, 1p/19q, and MGMT status) is presented in Sec. 3.1 and summarized in Table 1. This work comes from several independent institutions but remains preliminary, with limited external validation, and whether it can inform patient management has yet to be shown.

The value of detecting these alterations intraoperatively lies in what it could enable. Beyond providing a diagnosis, Raman spectroscopy could potentially support intraoperative tumor risk stratification based on molecular profiles. This could allow neurosurgeons to tailor their surgical approach considering tumor biology, location relative to eloquent structures, and patient performance status. For example, if validated in future studies, detection of CDKN2A/B homozygous deletion in an IDH-mutant astrocytoma could provide prognostic information relevant to the risk-benefit assessment of further resection, as this deletion indicates poor prognosis. However, this application remains exploratory.^57^

In addition to potential surgical benefits, Raman spectroscopy may help reduce treatment delays. If the technology can yield rapid preliminary molecular information intraoperatively, it could facilitate treatment planning while definitive sequencing results are pending, for example, identifying candidates for targeted therapies (such as IDH inhibitors) or clinical trials earlier.^112^ However, the clinical impact of such accelerated preliminary information on treatment outcomes has not been studied.

Raman spectroscopy as a treatment planning aid could be particularly useful for brain metastases. Research has demonstrated that mutation profile differences exist between a brain metastasis and its primary tumor.^113^ However, repeat mutation profile testing is not routinely performed on brain metastasis tissue if a mutation profile has been obtained from the primary tumor. If validated, Raman spectroscopy could potentially detect spectral changes associated with treatment-resistant mutations or novel mutations absent in the primary tumor, though this application remains speculative and has not yet been investigated in dedicated studies.

### 4.3 Comparison with Alternative Technologies

#### 4.3.1 Confocal laser endomicroscopy: real-time optical histology

Confocal laser endomicroscopy (CLE) is one of the most direct alternatives to Raman spectroscopy for intraoperative tissue assessment. This technology most commonly uses fluorescent contrast agents and laser to produce real-time digital images of tissue at cellular resolution, essentially providing "optical biopsies" during surgery. Using an *ex vivo* approach, CLE has demonstrated higher accuracy than frozen sections (89% versus 86%) in less time (13 min versus 28 min).^114^ *In vivo* CLE with intravenous fluorescein has also been demonstrated and, in a direct comparison, showed higher sensitivity than the *ex vivo* approach.^115^

CLE and Raman spectroscopy provide complementary rather than equivalent information: CLE produces morphological images equivalent to histology, whereas Raman reports molecular composition. Both are nondestructive, and each exists as an *in vivo* and *ex vivo* implementation that images in or near real time. CLE most often uses fluorescein, although label-free CLE based on tissue autofluorescence has been demonstrated, including *in situ* in brain tumors,^116^ whereas Raman typically operates label-free. At glioma margins, CLE-experienced neurosurgeons and neuropathologists show comparable diagnostic performance, though interpretation is subjective and specificity is limited in these infiltrative regions,^117^ whereas Raman classification is automated.^55^ FDA-cleared CLE systems are *in vivo* neurosurgical use,^118^ while Raman spectroscopy remains under investigational authorization.

#### 4.3.2 iKnife mass spectrometry: destructive but informative

The iKnife uses rapid evaporative ionization mass spectrometry to analyze surgical smoke during electrocautery, providing real-time tissue classification during tumor removal. Van Hese et al. demonstrated 88% accuracy in distinguishing tumor from normal brain with results available within seconds of tissue vaporization.^119^ The system is designed to integrate with standard electrosurgical instruments without additional surgical steps, while the molecular sensitivity of mass spectroscopy enables detection of specific metabolites and lipids that distinguish tumor types.

The iKnife’s fundamental limitation is its destructive nature: tissue must be vaporized to generate data, precluding assessment near critical structures. In contrast, Raman spectroscopy’s nondestructive assessment enables evaluation of functional areas without tissue damage, repeated measurements for confirmation, and comprehensive margin assessment, including critical structures, all before tissue resection.

#### 4.3.3 Emerging molecular approaches

Beyond optical and spectrometric techniques, rapid sequencing platforms such as Oxford Nanopore long-read sequencing show promise for intraoperative molecular characterization, with the potential to provide definitive genetic information within the surgical time frame.^120^ However, these approaches remain investigational, with validation largely limited to single-center studies.^121^ The technologies discussed in this section each occupy a different position on the trade-off between speed, tissue preservation, molecular depth, and clinical readiness, and the optimal intraoperative toolkit may combine several complementary approaches. Table 3 summarizes the key characteristics and current evidence level for each of these intraoperative technologies alongside the established structural adjuncts (iMRI and iUS).

**Table 3:** Comparison of intraoperative brain tumor assessment technologies.

Feature	Raman Spectroscopy	Fluorescence-Guided Surgery (5-ALA, Fluorescein)	Intraoperative Ultrasound	Intraoperative MRI	CLE	REIMS (iKnife)
Tissue preservation	Nondestructive	Nondestructive	Nondestructive	Nondestructive	Nondestructive *in vivo* imaging; *Ex vivo* also reported^118^	Destructive (vaporization)
Time to result	∼2 s per point^52^	Real-time (continuous)	Real-time (continuous)	20 to 60 min per scan^35^^,^^41^	Real-time imaging (∼0.1 s per frame); interpretation in minutes^114^	Within seconds^119^
Spatial coverage	Point sampling	Wide-field (entire cavity)	Wide-field (entire cavity)	Whole brain (volumetric)	Small field of view	Point sampling
Molecular information	Spectral fingerprint (*in vivo*); emerging molecular classification (IDH, 1p/19q; *ex vivo* only)	Fluorescence signal only (no molecular specificity)	Anatomical (residual tumor detection)	Anatomical (residual tumor detection)	Morphology only (no molecular)	Metabolite/lipid profiles
Reagent/agent required	None (label-free)	Oral 5-ALA (2 to 4 h pre-op) or IV Fluorescein (intra-op)^47^	None	Gadolinium (optional)	Fluorescein (IV); label-free demonstrated^116^	None
Operator dependence	Automated classification	Subjective visual assessment	Highly operator-dependent acquisition and interpretation^36^	Radiologist and surgeon interpretation	Neurosurgeon-interpretable with training; comparable to neuropathologist^117^	Automated classification
Low-grade glioma utility	Limited early evidence^55^	Limited; 5-ALA poor fluorescence uptake^43^ and fluorescein patchy in nonenhancing glioma^45^	Detects tumor bulk; infiltrative margins difficult to delineate (edema mimics tumor)^36^	Improved GTR rates^39^^,^^41^	Feasibility demonstrated in low-grade gliomas^122^	Classification demonstrated in grade II gliomas^119^
Workflow disruption	Minimal (<5 s per measurement)	Minimal (integrated with microscope)	Minimal (low cost, easily integrated, repeatable)	Substantial (dedicated suite, prolonged operative time) ^35^	Minimal for *in situ* imaging; moderate for *ex vivo* (biopsy required)	Minimal
Evidence level	Analytic validity (multicenter)^52^	5-ALA – Phase III RCT^106^	Meta-analysis + RCT: higher extent of resection in high-grade glioma^37^^,^^38^	RCTs for extent of resection^40^^,^^41^	Prospective multicenter pilot^114^	Single-center validation^119^
		Fluorescein – phase II prospective^47^				
Regulatory status	Health Canada Investigational Testing Authorization	5-ALA – FDA approved (Gleolan), EMA approved	Established; widely adopted clinical adjunct	FDA approved, implemented in specialized centers	FDA-cleared for neurosurgical imaging^115^^,^^118^	Research use only
		Fluorescein – off-label for HGG^46^				

Abbreviations: CLE, confocal laser endomicroscopy; 5-ALA, 5-aminolevulinic acid; REIMS, rapid evaporative ionization mass spectrometry; RCT, randomized controlled trial; FDA, Food and Drug Administration; EMA, European Medicines Agency; IV, intravenous; HGG, high-grade glioma.

## 5 Current Limitations and Technical Challenges

While the evidence discussed demonstrates promise, several barriers must be addressed before Raman spectroscopy can become a routine intraoperative tool.

### 5.1 Spectral Acquisition Challenges

Brain tissue contains multiple endogenous fluorophores (including NADH, flavins, lipopigments, and porphyrins)^123^ that produce broad fluorescence backgrounds potentially much stronger than Raman signals.^124^ This can obscure subtle spectral peaks needed for tissue discrimination and molecular diagnosis, particularly during *in vivo* measurements where blood contamination at the resection surface can further attenuate signal quality, though this is controlled with routine surgical field preparation. Selection of near-infrared excitation wavelengths (785 to 1064 nm) reduces fluorescence generation, while computational techniques, including polynomial baseline correction, derivative spectroscopy, and wavelet transformation, can isolate Raman signals after acquisition.^124^^,^^125^

Operative room lighting presents an additional challenge. Surgical microscope illumination and fluorescent ceiling lights create spectral artifacts that must be eliminated. Operative lights and neuronavigation infrared sources can be managed by directing them away from the measurement site.^126^ In practice, Raman acquisition requires a brief (<5  s) cessation of microscope illumination combined with background spectral subtraction.^75^ This protocol was integrated into a multicenter study workflow without significant surgical disruption.^52^

### 5.2 Machine Learning Model Generalizability and Dataset Limitations

The diagnostic accuracy of Raman spectroscopy depends on machine learning models trained on spectral datasets. A review found that studies splitting data at the spectra level reported consistently higher accuracies than those using patient-level splits, with independent test set performance falling substantially below cross-validated estimates in several cases.^127^ To date, few of the reviewed studies trained or tested across multiple instruments. A model trained on one spectrometer can transfer poorly to another because of calibration, intensity, and fluorescence differences. This raises concerns about overfitting to site-specific spectral characteristics arising from differences in instrumentation, tissue handling, or patient demographics.^127^ The only multicenter study was Ember et al., where predictive performances were reported using a machine-learning model trained in one center applied to measurements made in another center acquired by a different surgical team with a different instrument.^52^ Leblond *et al.* tested model generalizability to unseen tumor types, achieving positive predictive values of 70% for astrocytoma and 74% for oligodendroglioma.^55^ Class imbalance further degrades external validation, as demonstrated by the reduced performance for underrepresented molecular markers reported by Liu *et al.* and discussed earlier in Sec. 3.1.1.^57^ No public brain tumor Raman spectral benchmark dataset currently exists, limiting the ability to compare methods across groups.^127^ The validation target itself is also moving as the WHO CNS tumor classification is regularly revised.^10^ Classification models trained under earlier criteria may not align cleanly with the current diagnostic criteria, requiring revalidation as the classification is updated.

### 5.3 Cross-Institutional Reproducibility and Standardization

Hardware variability between Raman systems complicates cross-institutional reproducibility. An FDA evaluation found that sensitivity ranged from 37 to 1590 counts/mW across three fiber-optic Raman devices, concluding that specifications alone are insufficient to predict *in vivo* performance.^128^ A cross-laboratory study comparing identical samples across 15 institutions demonstrated inter-setup variability in peak positions, intensities, and signal-to-noise ratio, with standard wave number and intensity calibration only partially reducing these differences.^129^ Current Raman calibration standards covering instrument performance (ASTM E1683), wave number calibration (ASTM E1840), and spectral response correction (ASTM E2911) were developed for general analytical applications and do not fully address biomedical *in vivo* requirements.^128^ No consensus protocol for intraoperative Raman acquisition currently exists, though open-source preprocessing pipelines validated across multiple platforms offer a partial solution.^130^ Standardization of sampling protocols is equally important, as current studies report accuracy at the measurement level while clinical decisions require patient-level diagnostic accuracy with standardized decision thresholds.^127^

These reproducibility problems are compounded by the lack of standardization across probe systems. The *in vivo* and *ex vivo* probe studies discussed earlier differ in excitation wavelength, spot size, and contact geometry that define how much tissue each measurement samples, and in laser power and integration time. A spectrum and the accuracy figure attached to it do not transfer between systems, making performance figures reported by different groups difficult to compare directly.

### 5.4 Clinical Consequences of Diagnostic Errors

The consequences of diagnostic errors in neurosurgery are asymmetric and vary by location. A false positive (reporting a normal brain as a tumor) near the eloquent cortex could prompt unnecessary resection with a permanent neurological deficit. A false negative (reporting the tumor as normal brain) would leave the tumor behind, reducing the extent of resection.

Point-based spectroscopy also introduces another challenging error type. Because the probe samples discrete spots rather than the whole cavity, a false negative may reflect a sampling point miss as much as a classifier error: the residual tumor tissue was never sampled. Unlike wide-field adjuncts (5-ALA or iUS), Raman cannot currently map an entire margin, and the sampling density needed for reliable coverage remains undefined. This sparse sampling creates a trade-off between acquisition speed and the risk of missed infiltration.

The clinical implications of these errors depend on how surgeons integrate Raman information and whether it is used as a stand-alone decision tool or as an adjunct to established intraoperative methods. Published studies have not systematically characterized error rates^127^ or clinical consequences; prospective trials examining how Raman-derived information changes surgical decisions and patient outcomes are essential.

### 5.5 Regulatory Pathway and Clinical Translation

No Raman-based diagnostic device for brain tumor detection is currently marketed in the United States, ^128^ and the absence of a predicate device implies a more demanding approval pathway [e.g., De Novo or premarket approval versus 510(k)]. Molecular diagnostic claims (IDH status, 1p/19q codeletion, and MGMT methylation) would carry a higher regulatory burden than tissue detection claims, requiring clinical validity comparable to established molecular tests. The FDA approval of 5-ALA took a decade longer than European approval, illustrating the potential timeline challenges.^131^ Health Canada Investigational Testing Authorization has been obtained for one handheld probe system, which is undergoing multicenter clinical investigation,^52^ but this represents an early phase of the regulatory process. Progression toward routine clinical use will require multi-institutional trials designed to meet regulatory evidentiary standards. Cost-effectiveness within existing surgical infrastructure also requires formal evaluation, particularly for resource-limited settings.

### 5.6 Path Forward

Sample sizes remain limited for the *in vivo* and spontaneous-Raman work: the largest intraoperative spontaneous Raman study enrolled 67 patients, and the *ex vivo* spontaneous-Raman molecular studies have mostly involved fewer than 50. SRH-based imaging studies are larger (153 patients in the DeepGlioma validation and a 967-patient training set in the SIGNAL preprint), though these rest on a single commercial platform and are *ex vivo*. Early independent prototypes are beginning to appear, including a portable full-spectrum SRS system tested against frozen section in glioblastoma, though so far only at the feasibility level.^132^ Several converging needs have been identified: large-scale prospective trials with survival or clinical decision-making endpoints; public benchmark Raman spectral datasets to enable standardized method comparison;^127^ and prospective randomized comparisons against established adjuncts, including 5-ALA fluorescence-guided surgery, iUS, and iMRI. In addition, critical optical parameters such as laser power at tissue and irradiance are inconsistently reported across studies, with few explicitly referencing safety standards (e.g., ANSI Z136 and IEC 60825). Similarly, accuracy metrics are frequently reported as point estimates, and confidence intervals and detailed cohort demographic information are not consistently provided. Until these gaps are addressed, Raman spectroscopy should still be regarded as an investigational adjunct rather than a tool for routine clinical use in neurosurgical oncology.

## 6 Outlook

If the validation requirements outlined in Sec. 5 are addressed, Raman spectroscopy could evolve from a margin-detection adjunct into a tool for providing real-time biochemical information at the point of surgical decision-making. Its use alongside existing tools could support risk-stratified resection and earlier identification of candidates for targeted therapies as molecular classification becomes increasingly central to clinical management. Realizing this potential requires prospective trials demonstrating patient benefit, not only diagnostic accuracy. Across the field, clinical translation is running ahead of the implementation evidence: diagnostic-accuracy studies and even commercial systems are accumulating, while the health-economic and workflow-integration case for intraoperative Raman in neurosurgery remains largely unexplored.

## Data Availability

This review article contains no new data or code generated or analyzed. All data discussed are available in the cited publications.
